# Repetitive transcranial magnetic stimulation (rTMS) for treatment-resistant major depression (TRMD) Veteran patients: study protocol for a randomized controlled trial

**DOI:** 10.1186/s13063-017-2125-y

**Published:** 2017-09-02

**Authors:** Zhibao Mi, Kousick Biswas, J. Kaci Fairchild, Anne Davis-Karim, Ciaran S. Phibbs, Steven D. Forman, Michael Thase, Gerald Georgette, Tamara Beale, David Pittman, Margaret Windy McNerney, Allyson Rosen, Grant D. Huang, Mark George, Art Noda, Jerome A. Yesavage

**Affiliations:** 1 Department of Veterans Affairs, Cooperative Studies Program Coordinating Center, Perry Point, MD USA; 2 Department of Veterans Affairs, Sierra-Pacific MIRECC, and WRIISC, Palo Alto, CA USA; 3 Department of Psychiatry and Behavioral Sciences, Stanford University School of Medicine, Stanford, CA USA; 4 Department of Veterans Affairs, Cooperative Studies Program Pharmacy Coordinating Center, Albuquerque, NM USA; 5 Department of Veterans Affairs, VA Medical Center, Pittsburgh, PA USA; 6 Department of Veterans Affairs, VA Medical Center, Philadelphia, PA USA; 7 Department of Veterans Affairs, Cooperative Studies Program Central Office, Washington DC, USA; 8 Department of Veterans Affairs, Ralph H. Johnson VA Medical Center, Charleston, SC USA; 9 Brain Stimulation Laboratory (BSL), Psychiatry Department, Medical University of South Carolina (MUSC), Charleston, SC USA; 10 Western Psychiatric Institute and Clinic, University of Pittsburgh School of Medicine, Pittsburgh, PA USA; 11 Department of Pediatrics, Stanford University School of Medicine, Stanford, CA USA; 12 VISN21 MIRECC, Department of Veterans Affairs, Department of Psychiatry, Stanford University School of Medicine, 3801 Miranda Avenue, Palo Alto, CA 94304 USA

**Keywords:** Repetitive transcranial magnetic stimulation (rTMS), Treatment-resistant major depression (TRMD), Hamilton Rating Scale for Depression (HRSD24)

## Abstract

**Background:**

Evaluation of repetitive transcranial magnetic stimulation (rTMS) for treatment-resistant major depression (TRMD) in Veterans offers unique clinical trial challenges. Here we describe a randomized, double-blinded, intent-to-treat, two-arm, superiority parallel design, a multicenter study funded by the Cooperative Studies Program (CSP No. 556) of the US Department of Veterans Affairs.

**Methods:**

We recruited medical providers with clinical expertise in treating TRMD at nine Veterans Affairs (VA) medical centers as the trial local investigators. We plan to enroll 360 Veterans diagnosed with TRMD at the nine VA medical centers over a 3-year period. We will randomize participants into a double-blinded clinical trial to left prefrontal rTMS treatment or to sham (control) rTMS treatment (180 participants each group) for up to 30 treatment sessions. All participants will meet *Diagnostic and statistical manual of mental disorders, 4* ^*th*^ *edition* (DSM-IV) criteria for major depression and will have failed at least two prior pharmacological interventions. In contrast with other rTMS clinical trials, we will not exclude Veterans with posttraumatic stress disorder (PTSD) or history of substance abuse and we will obtain detailed history regarding these disorders. Furthermore, we will maintain participants on stable anti-depressant medication throughout the trial. We will evaluate all participants on a wide variety of potential predictors of treatment response including cognitive, psychological and functional parameters.

**Discussion:**

The primary dependent measure will be remission rate (Hamilton Rating Scale for Depression (HRSD24) ≤ 10), and secondary analyses will be conducted on other indices. Comparisons between the rTMS and the sham groups will be made at the end of the acute treatment phase to test the primary hypothesis. The unique challenges to performing such a large technically challenging clinical trial with Veterans and potential avenues for improvement of the design in future trials will be described.

**Trial registration:**

ClinicalTrials.gov, NCT01191333. Registered on 26 August 2010. This report is based on the protocol version 4.6 amended in February 2016. All items from the World Health Organization Trial Registration Data Set are listed in Appendix A.

**Electronic supplementary material:**

The online version of this article (doi:10.1186/s13063-017-2125-y) contains supplementary material, which is available to authorized users.

## Background

Major depressive disorder (MDD) is prevalent in about 10% of American medical outpatients in any given year [1]. As many as 20% of these patients respond incompletely, or do not respond at all, to successive trials of multiple classes of antidepressant and mood stabilization medications and psychotherapy [2, 3]. Thus, within the Veterans Affairs (VA) population, there are roughly 100,000 patients with treatment-resistant major depression (TRMD). In such cases, the general treatment strategy is usually to advance treatment delivery in a way that increases response rates, albeit at the expense of increased risks and increased side effects. One example would be the use of monoamine oxidase inhibitors (MAOIs). Another preferred treatment modality for TRMD is electroconvulsive therapy (ECT) [1, 4]. However, despite being the most effective antidepressant in the acute setting, ECT usage is limited by post-treatment amnesia and confusion, the medical risks of general anesthesia, the high costs associated with inpatient hospitalization, general apprehension about the procedure among candidate patients, and some administrative impediments. Such approaches may be reasonable for those depressed patients who are suicidal or who have the most severe symptoms. However, for the majority of patients with TRMD whose symptoms are moderate, the decision to escalate treatments is more difficult. Thus, new TRMD treatments are needed, preferably without major safety concerns or side effects as seen with aggressive polypharmacy or ECT.

### Repetitive transcranial magnetic stimulation (rTMS) in the treatment of major depression

rTMS is a method of delivering brain stimulation with neither the seizures or risks associated with ECT, nor the potential side effects and risks of pharmacological augmentation strategies, such as MAOI therapy. It may offer a viable alternative to ECT for some patients. Several studies, including a systematic review and meta-analysis of the studies to date, appear to show a positive effect in TRMD [5] with an effect size of Cohen’s *d* of about 0.65 (moderate effect). This is comparable with that of contemporary antidepressant medications. The most recent large-scale clinical trial using advanced coil designs documented an effect size of 0.76 [6]. With a minimal side-effect profile, and the rarity of untoward events and side effects [5], safety concerns about the use of rTMS are considerably fewer than for ECT. Importantly, rTMS may be less expensive to administer than ECT (largely due to not requiring anesthesia) [7]. Thus, there is the potential for a significant advance in VA mental health care, with associated cost savings, if rTMS were to be shown effective in treating TRMD in VA patients.

### Importance of efficacious anti-depressant treatment in Veteran populations

Despite these positive studies of rTMS in civilian populations, research suggests that Veterans may experience a differential response to mental health treatments compared to the response seen in civilians. This finding has been well-documented in studies of treatment of posttraumatic stress disorder (PTSD) in which Veterans did not show evidence of the treatment gains seen in civilians in both pharmacological and psychotherapy trials [8, 9]. The reasons for this treatment disparity are most likely multi-factorial in nature. Clinical trials of new antidepressant treatments are initially tested in highly selected patients, free from comorbid conditions, and not taking other antidepressant medications. Yet, compared to civilian populations, Veterans experience a greater preponderance of medical and psychiatric comorbidities that complicate their clinical presentation and negatively impact their response to therapeutic intervention [10]. For example, in the national Veteran population that carries a diagnosis of a depressive disorder (N = 946,342 in the 2005 outpatient file), over 80% have at least one additional psychiatric diagnosis with the most common dual-diagnoses being PTSD (39%) and substance use disorder (45%). Most of these patients are already taking one or more psychotropic drugs. Furthermore, the VA has a special concern about treating potentially suicidal Veterans. Thus at this time, it is not clear if the results from the positive rTMS studies in civilian populations will translate into similar effects in the VA practice setting with all its comorbidities, multiple medications and risk for impulsive behavior. Given the fixed resources available for mental health treatment in the VA, rational allocation of resources is best guided by clinical trials in the exact population in which the treatments will be utilized, which led to several challenges in study design.

## Methods/design

A specialized rTMS clinical trial in Veterans must satisfy several conditions. These include selection of a representative population of Veterans with TRMD with appropriate consideration of VA-centric mental health comorbidities (e.g., PTSD, substance use disorders, and suicidality), careful development of a control (sham) rTMS procedure, and attention to moderators and mediators of treatment response. Thus, we designed the trial to enroll 360 Veterans diagnosed with TRMD at nine VA participating medical centers over a 3-year period. All nine participating medical centers have been reviewed and approved by the VA Central Institutional Review Board (CIRB) with the reference number of 10-08. We randomize all participants in a double-blinded clinical trial to left prefrontal rTMS treatment or to sham (control) rTMS treatment (180 participants each group) for up to 30 treatment sessions. We evaluate all participants on a wide variety of measures including cognitive, psychological, and functional parameters. The primary dependent measure will be remission rate (Hamilton Rating Scale for Depression (HRSD24) ≤ 10), and secondary analyses will be conducted on other indices including economic measures.

We follow the NIH protocol procedures for administration and certification of the HRSD ratings. This will include the use of a prepared script to help administer the HRSD. Certification of all raters at a participating site will be verified prior to enrollment. This will be done by shipping recordings of mock interviews (non-patient) to the sites where trained raters have determined a “gold standard HRSD score”. Site raters will then submit their scores. Following NIH procedures, large deviations will be noted, and a rater can have an additional test. This can be repeated for a total of three times until the site is told they must find another rater.

Following NIH procedures, to ensure that HRSD do not “drift” over time, one HRSD recording will be circulated to evaluators at all participating sites every 6 months. The evaluators will be asked to rate this recording and to return their ratings. Evaluators who drift by more than 3 points on the HRSD total score will receive a telephone consultation followed by one additional HRSD recording.

**Fig. 1:**
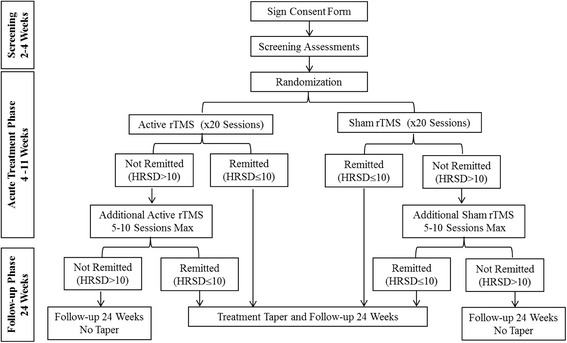
Study flow chart. *rTMS* repetitive transcranial magnetic stimulation, *HRSD* Hamilton Rating Scale for Depression

**Fig. 2:**
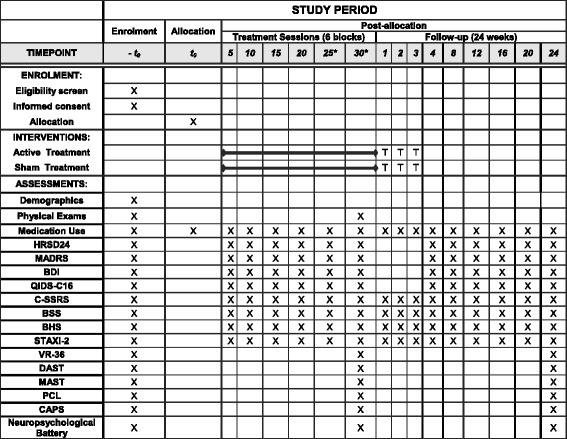
Standard Protocol Items: Recommendations for Interventional Trials (SPIRIT): the schedule of enrollment, interventions, and assessments. *HRSD* Hamilton Rating Scale for Depression, *MADRS* Montgomery-Asberg Depression Rating Scale, *BDI* Beck Depression Inventory, *QIDS-C16* Quick Inventory of Depressive Symptomatology, *CSSRS* Columbia Suicide Severity Rating Scale, *BSS* Beck Scale for Suicide Ideation, *BHS* Beck Hopelessness Scale, *STAXI-2* State Trait Anger Expression Inventory-2, *VR-36* Veterans RAND 36 Item Health Survey, *MAST* Michigan Alcohol Screening Test, *DAST* Drug Abuse Screening Test, *PCL* Posttraumatic Stress Disorder Checklist, *CAPS* Clinician Administered PTSD Scale, *T* Treatment taper

We will compare the rTMS and the sham groups at the end of the acute treatment phase to test the primary hypothesis. The study will last 3.5 years, with a 3-year enrollment period. Participants engage in study procedures for a total of approximately 30–39 weeks (2–4 weeks of screening, 4–11 weeks of the acute treatment phase and the 24-week follow-up phase). The flow diagram (Fig. 1) provides an overview of the design and procedures. The Standard Protocol Items: Recommendations for Interventional Trials (SPIRIT) study schedule is detailed in Fig. 2, and the SPIRIT checklist is shown in Additional file 1.

### Population

We define patients with TRMD as Veterans who meet the *Diagnostic and statistical manual of mental disorders, 4* ^*th*^ *edition* (DSM-IV) criteria for MDD and who have failed at least two prior pharmacological interventions as defined by a modified and updated version of the Antidepressant Treatment History Form (ATHF) [11]. We will not exclude Veterans with PTSD or history of substance use and we will obtain detailed history on these disorders. Participants will remain stable on their antidepressant medication regimen throughout rTMS treatment.

We designed the inclusion/exclusion criteria to identify patients with TRMD who exhibit a full range of the manifestations of that condition. Furthermore, we intend our recruited population to represent the VA pool of patients with TRMD. Table 1 shows the detailed inclusion/exclusion criteria.

**Table 1:** rTMS trial inclusion and exclusion criteria

Inclusion criteria	Exclusion criteria
Between 18 and 80 years of age.	Pregnant or lactating woman (this is an FDA-required exclusion. In the future, if rTMS becomes a proven treatment for major depression, its safety in the context of pregnancy should be studied separately)
Using the Structured Clinical Interview for DSM Disorders (SCID) for DSM-IV-TR patients will be diagnosed MDD	Unable to be safely withdrawn, at least 2 weeks prior to treatment commencement, from medications that substantially increase the risk of having seizures
Have a HRSD24 ≥ 20 no more than 7 days prior to randomization	Have a cardiac pacemaker
Exhibit moderate level of resistance to antidepressant treatment defined, using the ATHF, as failure of at least two adequate medication trials	Have an implanted device or metal in the brain
Duration of current episode of MDD ≤ 10 years	Have a cochlear implant
Ability to obtain a motor threshold (MT) (should be determined at the end of the screening process)	Have a mass lesion, cerebral infarct, increased intracranial pressure, or other active central nervous system (CNS) disease, including a seizure disorder
Currently under the care of a VA psychiatrist	Known current psychosis as determined by DSM-IV or SCID (axis I, psychotic disorder, schizophrenia) or history of a non-mood psychotic disorder
If on a psychotropic medication regimen, that regimen will be stable for at least 4 weeks prior to randomization in the study and patient will be willing to remain on a stable regimen during the acute treatment phase	Known current bipolar I disorder as determined by SCID or a history of bipolar I disorder
Has an adequately stable condition and environment to enable attendance at scheduled clinic visits	Current amnestic disorders, dementia, Blessed Orientation-Memory-Concentration score > 10, delirium, or other cognitive disorders
For female participants, agrees to use one of the following acceptable methods of birth control	Current substance abuse (not including caffeine or nicotine) as determined by positive toxicology screen, or by history via SCID, within 3 months prior to screening
Able to read, verbalize understanding and voluntarily sign the Informed Consent Form prior to performance of any study-specific procedures or assessments	Patients with an elevated risk of seizure due to TBI
	Participation in another concurrent clinical trial
	Patients with prior exposure to rTMS
	Active current suicidal intent or plan as evidenced by a score of 4 or 5 on the suicidal ideation portion of the Columbia Suicide Severity Rating Scale (CSSRS) or the endorsement of an actual attempt, interrupted attempt, or an aborted attempt in the past 6 months
	Unstable cardiac disease or recent (<3 months previous) myocardial infarction
	Patient refuses to sign consent for participation in the study

*SCID* Structured Clinical Interview for DSM Disorders, *MDD* major depressive disorder, *HRSD* Hamilton Rating Scale for Depression, *ATHF* Antidepressant Treatment History Form, *VA* Veterans Affairs, *FDA* Food and Drug Administration, *rTMS* Repetitive transcranial magnetic stimulation, *TBI* traumatic brain injury

Potential participants will be recruited through a number of methods. These include, but are not limited to, referral by primary providers, referral by mental health providers, flyers posted in common areas such as canteens at VA hospitals, review of the VA administrative databases containing information for both outpatient and inpatient encounters, which are housed in the Austin Information Technology Center, sending IRB-approved messages to providers twice a year, and posting basic information about the study in local VA SharePoint sites. Local study staff will work with providers at their respective medical centers to identify Veterans who may be appropriate for participation in this study. Although most recruitment will occur in mental health clinics, recruitment within women’s health clinics will be used to try to maximize the enrollment of eligible women.

### Randomization to treatment

The Site Investigator (SI) will obtain informed consent (Additional file 2). Patients who sign the Informed Consent Form and meet the study eligibility criteria will be enrolled into the study and will be randomized to either rTMS or sham rTMS. We use an adaptive randomization scheme to approximately equate numbers of patients randomized to each treatment group within several important subsets. These subsets include enrollment site and whether patients have a substance use disorder and/or PTSD. We will make treatment assignments using a “biased coin” procedure that should improve overall balance across subsets. We calculate imbalance by summing the marginal totals for these three factors for each treatment group and calculating the difference, D. If the imbalance is < 3, we will assign to rTMS or sham rTMS with equal probability; otherwise, we will assign to the group that increases imbalance with probability, 1/D. We incorporate this approach into our electronic data capture system.

To randomize a patient into the study, the SI or the Study Coordinator (SC) will submit the electronic randomization form. This computerized system, after verifying eligibility, will randomize a patient to either the rTMS or to the sham rTMS treatment group. A non-sequential treatment number will be assigned. This unique treatment number will be key-entered into the device which will be associated with a treatment assignment and will enable the rTMS device to deliver the appropriate treatment (active or sham) to each patient. Every attempt will be made to randomize a participant so that he/she will receive his/her first rTMS treatment as soon as possible after randomization.

### Blinding

As this is a double-blinded study, both participants and on-site study staff (including site investigators, site coordinators, clinicians administering study treatment, and staff involved in assessing adverse events and study outcomes) will be blinded to group assignment. At no point will participants or study staff be unblinded to group assignment prior to study completion. The Data Monitoring Committee (DMC) will determine when they should be unblinded to treatment assignment for the reviewing of adverse event data. A questionnaire will be used before and after the first treatment session and again at the end of the final study visit to elicit patient and study staff perception of group allocation.

### Safety and adverse events

During the course of this study, all study procedures will adhere to the most current safety and application guidelines for administration of rTMS [12]. We will monitor adverse events per VA and US Food and Drug Administration (FDA) regulations. However, due to the complex psychiatric presentation of the typical VA patient with TRMD, we have developed additional safety protocols for the monitoring of suicidality and substance use. Study personnel will work closely with the participant’s primary mental health provider in situations in which increased suicidality or substance use is suspected to ensure full clinical coverage of these Veterans.

#### Suicidality monitoring protocol

While suicide risk is not the primary focus of this trial, the complex clinical presentation of VA patients with TRMD necessitates its close monitoring. All participants will participate in safety planning with study personnel prior to randomization. Study participants will also undergo a comprehensive suicidality battery at screening, at the end of each treatment block and taper week, and at all follow-up visits. Measures included in the suicidality battery are the Columbia Suicide Severity Rating Scale [13], Beck Scale for Suicide Ideation (BSS) [14], Beck Hopelessness Scale [15], State Trait Anger Expression Inventory-2 [16], and items from the primary and secondary outcome measures, HRSD24 and Montgomery-Asberg Depression Rating Scale (MADRS). If at any time a Veteran is determined to have a significant risk for suicide, study personnel with extensive training in the suicidality protocol suicidality assessment will appropriately intervene.

#### Substance use monitoring protocol

As substance use and abuse are associated with both increased risk of seizure and completed suicide, ongoing monitoring of substance use is an important component of this trial. All participants will undergo a urine toxicology screen and a breathalyzer test prior to study randomization and then randomly throughout the acute treatment, taper, and follow-up phases. In addition to these random assessments, study personnel will also monitor substance use with paper and pencil measures prior to study entry, at the end of each treatment block and taper week, and at all follow-up visits. Measures included in the substance use monitoring battery, include Michigan Alcohol Screening Test [17], Drug Abuse Screening Test [18], and the Drug and Alcohol Timeline Follow back methods [19]. Personnel administering rTMS will also ask participants if they have used any substances prior to all administrations of TMS. As with the suicidality protocols, study personnel will receive extensive training on the substance use monitoring protocol and will appropriately intervene at such time a participant has screened positive for substance use. To insure the validity and integrity of this clinical trial, all assessments and the rTMS treatment will only be delivered by trained study staff.

## Study management

### Data Coordinating Center

The Cooperative Studies Program Coordinating Center (CSPCC), located in Perry Point, MD, USA, will provide administrative, data management and statistical support for the study. CSPCC staff will provide guidance on completion of forms and data quality queries. They will develop editing software and manage the study database (Additional file 3). All reports generated during the ongoing phase of the study and the final statistical analyses will be the responsibility of the CSPCC.

### Executive Committee

The Executive Committee is the management and decision-making body for the operational aspects of the study. This committee is chaired by Dr. Jerome Yesavage, and includes the Study Biostatistician, the CRP, a minimum of three SIs, and outside consultants, if necessary. This committee monitors the performance of participating sites and the quality of data collected. The Executive Committee formulates plans for publications and oversees the publication and presentation of all data from the study. Permission from this committee must be granted before any study data may be used for presentation or publication. This group also does not receive outcome data during the course of the study. Executive Committee decisions that need to be made between regularly scheduled meetings will be made during periodic phone conferences.

### Data Monitoring Committee

The DMC is a group of outside experts in the area of TRMD, clinical trials and biostatistics that reviews the progress of the study and monitors patient enrollment, outcomes, adverse events, and other issues related to patient safety. The DMC makes recommendations to the CSP Director as to whether the study should continue or be modified or terminated. The DMC can consider patient safety or other circumstances as grounds for early termination, including either compelling internal or external evidence of treatment differences or unfeasibility of addressing the study hypotheses (e.g., poor patient intake, poor adherence to the protocol). The DMC will meet annually to review data reports prepared by the CSPCC. At the 6-month interval between the annual meetings, the DMC will receive a data report for their review. This group will receive outcome data during the course of the study. In order for the DMC to make its recommendation for continuation of the study, it will be necessary for them to see the analyses for the primary outcome measure every time that the report is run and it is possible to calculate the primary outcome measure. Periodic monitoring of interim results can significantly affect the probability of making an incorrect decision. A number of formal techniques have been developed for interpreting interim results. At the organizational meeting, the DMC will select the technique that it wants to use to monitor the study.

### Site Monitoring, Auditing and Resource Team (SMART)

The SMART will conduct monitoring visits to each participating site to monitor investigative records and practices to ensure sites are in compliance with both the study protocol and GCP. These site visits will occur annually or more frequently if directed by the study Monitoring Plan. Independent quality assurance audits will also be conducted at selected sites if needed.

### Central Institutional Review Board (CIRB)

The VA CIRB will approve and oversee the ethical and human subjects aspect of the study. This includes review of adverse events, the protocol and its amendments, and annual reports. All CIRB protocol amendment approvals are shared with the entire Study Team.

## Intervention

### Treatment stimulus

We will administer rTMS using a modified MagPro R30 device with Cool-B65-A/P coil. Active treatment will consist of 4000 pulses per session, delivered over the left prefrontal cortex at 120% of motor threshold (MT). We will determine MT at screening and at the beginning of every treatment block (every 5 sessions) with a separate C-B60 MT coil using electromyographic measurement of the resting right thumb (right abductor pollicus brevis). Threshold will be determined using an adaptive procedure (ClinicalResearcher software, Knoxville, TN, USA). Treatment will be delivered at a pulse frequency of 10 Hz, length of each pulse train of 4 seconds, time between pulse trains of 10 seconds, and length of treatment of 25 minutes for each treatment session. Units of five treatment sessions will be delivered over a minimum of 5 calendar days and a maximum of 12 calendar days. Patients will receive a minimum of 20 sessions of treatment and, for those not clinically remitting, up to a maximum of 30 sessions. In addition, participants meeting criteria for remission after 20–30 sessions will receive six additional taper treatments delivered over a 3-week period. Thus, patients will receive up to a total of 144,000 stimulation (sham or active) pulses in the study.

### Treatment control

The credibility of the study depends on the development of a highly believable sham treatment. Sham treatment will be accomplished by using the inactive side of the Cool-B65-A/P coil that functions both as an active (A) and placebo (P) coil. The coil is completely symmetrical and does not distinguish the A and P sides. Treatment condition (A vs P) is determined electronically in the software of the device, based on a predetermined random treatment assignment table hard-coded in each device’s memory and which is not accessible to study staff. The coil has a built-in position sensor and a symmetrical mechanical design with no markings that would differentiate the active or placebo sides. At the start of each session, a participant’s randomization number is entered into the device, triggering the device to instruct the TMS administrator on how to orient the coil based on a built-in position sensor and the treatment assignment (active/placebo) associated with the randomization number. Consequently, it is not possible for the operator to see or hear which side is used. Additionally, for each treatment session, whether sham or active, each patient will wear scalp electrodes through which a low-voltage, low electric current (2–6 mA at no more than 100 V) will be passed in order to provide cutaneous stimulation that mimics the sensation of actual rTMS. At the same time, we use a stimulation-synchronized white noise generator to mask the “click” noise from each stimulation. Both the patient and the treatment administrator receive the masking noise. We make every attempt to well-mask the patient, the treatment administrator, and all personnel at each treatment site to the treatment group assignment.

### Stimulation site: the 6 cm rule

We define the standardized treatment location over the left dorsolateral prefrontal cortex by moving the TMS coil 6 cm anterior to the MT location along the left superior oblique plane with a rotation point about the tip of the patient’s nose [20–22]. Our decision to move 6 cm rather than 5 cm reflects an evolution over several clinical trials. The optimal stimulation site, the dorsolateral prefrontal cortex is believed to be in Brodmann’s areas 9 and 46 and the vast majority of TMS studies move 5 cm rather than 6 cm anterior from the MT site. Traditionally this approach is called the “5 cm rule”. Unfortunately, a previous MRI study found that in a substantial number of patients the location was in the premotor rather than dorsolateral prefrontal cortex. Furthermore, these patients in whom the localization was inaccurate had an inferior treatment response [23]. The OP-TMS trial adopted a hybrid approach to targeting using this 5 cm rule [24] but also performing MRI and moving 1 cm anterior, to adjust for patients in whom there was inappropriate localization to the premotor cortex [25]. This hybrid approach led to fewer patients with inaccurately selected targets and an associated inferior treatment response. This current study adopts a 6 cm rule but also performs a separate study using MRI in patients from six of the nine sites for verification of accuracy and effect on treatment response (VA Funding CS R&D CX000604). Stimulation locations will also be related to potential functional MRI targets to assess this rule as an alternative to image guidance in patients in whom MRI is not feasible. Consistency in targeting across TMS sessions and in evaluating the accuracy in the MRI will be accomplished by marking the stimulation on a cloth cap (Magventure).

A separately funded MRI protocol (see above) will collect additional data on the 6 cm rule as implemented here. That study will use T1-weighted, structural MRI from the Alzheimer’s Disease Neuroimaging Initiative (ADNI) to compute the accuracy of the location using the TMS stimulation location marked with a fiducial marker as in Herbsman et al. [23]. This T1 structural image will also allow measurement of the distance of TMS coil from the cortex (skull-to-cortex distance). This variable has been demonstrated to be an important potential moderator of treatment response because there is a substantial decrease in the strength of the magnetic field with increased distance of the coil from the brain in older patients with brain atrophy [26]. There has also been success with targeting based on image guidance [27], so that both active and resting state fMRI protocols from the functional Brain Imaging Research Network are being collected [28] and relative proximity of the 6 cm rule to these alternative targets will be computed.

The patient’s participation in the study may be terminated at any time if the SI deems that the patient has not been following the protocol. This will generally be done only when the protocol violation significantly increases the risk associated with continuing to participate in the study. Any female participant who becomes pregnant during the acute treatment phase of the study will discontinue the study treatments for safety reasons, as the effects of rTMS on the unborn fetus is not known at this time, and she will immediately enter the follow-up phase. Any female participant who becomes pregnant during the follow-up phase of the study will continue to be followed up in accordance with the protocol and will complete all assessments.

## Assessment and analysis

### Outcomes

The primary outcome for this trial will be remission as defined by a score of 10 or less on the HRSD24. This HRSD is widely considered the “gold-standard” depression measure and is the most widely used assessment of depression in rTMS trials. Secondary outcomes will include reduction in depression as measured by the Montgomery-Asberg Depression Rating Scale (MADRS), reduction in suicide ideation as measured by the Beck Scale for Suicide Ideation (BSS), depression as measured by the Beck Depression Inventory (BDI), quality of life as assessed by the Veterans RAND 36-Item Health Survey (VR-36), and cognitive function as measured by a neuropsychological battery. The selection of these measures was based upon their use in previous studies of rTMS in TRMD. The detailed outcome collection scheme and data elements are shown in Tables 2 and 3.

**Table 2:** Study efficacy outcome measures

Outcomes		Metric scale	Schedule
Primary outcome	Hamilton Rating Scale for Depression (HRSD)	24-Item instrument with overall score range of 0–76	Measured at screening phase as baseline, end of each acute treatment week, and end of every 4 weeks during the follow-up phase
Secondary outcomes	Montgomery-Asberg Depression Rating Scale (MADRS)	10-Item instrument with overall score range of 0–60	Measured at screening phase as baseline, end of each acute treatment week, and end of every 4 weeks during the follow-up phase
	Beck Depression Inventory (BDI)	21-Item self-report test with overall score range of 0–63	Measured at screening phase as baseline, end of each acute treatment week, and end of every 4 weeks during the follow-up phase
	Beck Scale for Suicide Ideation (BSS)	21-Item self-report test with overall score range of 0–38 with last 2 items not counted in scoring	Measured at screening phase as baseline, end of each acute treatment week, end of each taper week and week 4, and end of every 4 weeks during the follow-up phase
	Veterans RAND 36-Item Health Survey (VR-36)	A self-administered survey consisting of two parts, i.e. Physical Component Summary (PCS, standardized score range of 0–100) and Mental Component Summary (MCS, standardized score range of 0–100)	Measured at screening phase as baseline, end of the acute treatment phase, and end of the follow-up phase
	Neuropsychological Battery	A cognitive function test including measures of executive function, attention, memory, visuospatial ability, processing speed, psychomotor function, and premorbid intelligence	Measured at screening phase as baseline, end of the acute treatment phase, and end of the follow-up phase

**Table 3:** Study performance and data element assessment scheme

Assessment (domain) and specific measurement	Weeks 2–4	Acute treatment phase						Follow-up phase								
		4–11 weeks						24 weeks								
		End of session number^a^						Taper			Follow up					
		5	10	15	20	[25]	[30]	1	2	3	4	8	12	16	20	24
Consent	S*															
Screening	S															
Randomization	B*															
Baseline	S															
Medical history	S															
Physical exam	S						x									
Laboratory measurements	S															
SCID-I	S															
Current/past ATHF	S															
Lifetime drinking history	B															
CAPS	B						x									x
THQ	B															
LSC-R	B															
BOMC	S															
Medication use	S	x	x	x	x	x	x	x	x	x	x	x	x	x	x	x
rTMS treatment log		x	x	x	x	x	x									
rTMS taper log								x	x	x						
HRSD and MADRS	S^b^	x	x	x	x	x	x				x	x	x	x	x	x
BDI	B	x	x	x	x	x	x				x	x	x	x	x	x
QIDS-C16	B	x	x	x	x	x	x				x	x	x	x	x	x
C-SSRS	S															
C-SSRS follow up		x	x	x	x	x	x	x	x	x	x	x	x	x	x	x
BSS	S	x	x	x	x	x	x	x	x	x	x	x	x	x	x	x
BHS	S	x	x	x	x	x	x	x	x	x	x	x	x	x	x	x
VR-36	B						x									x
Neuropsychological battery	B						x									x
DAST	S						x									x
PCL	B						x									x
MAST	S						x									x
STAXI-2	S	x	x	x	x	x	x	x	x	x	x	x	x	x	x	x
Urine toxicology screen and alcohol test	S		x		x		X		x		x		x		x	
Protocol deviation	As required															
AE/SAE/UADE	As required															

Note, for primary analyses and descriptive statistics total scores are used. *Abbreviations*: *SCID* Structured Clinical Interview for DSM Disorders, *ATHF* Antidepressant Treatment History Form, *CAPS* Clinician Administered PTSD Scale, *THQ* Trauma History Questionnaire, *LSC-R* Life Stressor Checklist-revised, *BOMC* Six-Item Blessed Orientation-Memory-Concentration, *rTMS* Repetitive transcranial magnetic stimulation, *HRSD* Hamilton Rating Scale for Depression, *MADRS* Montgomery-Asberg Depression Rating Scale, *BDI* Beck Depression Inventory, *QIDS-C16* Quick Inventory of Depressive Symptomatology, *C-SSRS* Columbia Suicide Severity Rating Scale, *BSS* Beck Scale for Suicide Ideation, *BHS* Beck Hopelessness Scale, *VR-36* Veterans RAND 36-Item Health Survey, *DAST* Drug Abuse Screening Test, *PCL* Posttraumatic Stress Disorder Checklist, *MAST* Michigan Alcohol Screening Test, *STAXI-2* State Trait Anger Expression Inventory-2, *AE* adverse events, *SAE* serious adverse events

*B Baseline

*S Screening

^a^Sessions 21–25 and 26 − 30 may not be required if patient goes into remission earlier

^b^Must be conducted within 7 days prior to randomization

There is growing evidence to suggest there are neurocognitive benefits of rTMS in TRMD. Specifically, improvements in verbal memory were demonstrated, seemingly independent of the antidepressant effects of rTMS. Additional improvements in verbal learning and psychomotor speed are also evidenced, though these are more likely the result of reduction of depressive symptoms [29, 30]. Based on these results, a brief neuropsychological battery was selected to examine as secondary outcomes in the present trial. Measures included in this battery include the Rey Auditory-Verbal Learning Test (RAVLT), Trail Making Test (TMT), Symbol Digit Modalities Test (SDMT), Judgement of Line Orientation (JLO), Controlled Oral Word Association Test (COWAT), Stroop Color and Word Test, and the North American National Adult Reading Test (NAART).

### Economic analysis

The economic analysis will take the perspective of the VA health care system, and include both short-term and longer-term analyses. All analyses will follow current standards for economic analyses of healthcare interventions [31–33]. Study data will be used to determine rTMS treatment costs, and VA production costs will be used for all other treatment costs. The main outcome for the economic analysis will be the primary study outcome, but the Short Form 36-Item Health Survey for Veterans (SF-36V) will be converted to utilities to allow comparisons to the cost-effectiveness of interventions for other conditions. The short-term analyses will use study data and VA databases to follow subjects for up to a year. Decision modeling will be used to extend the analysis to a lifetime. A budget impact analysis will also be conducted to facilitate VA management decisions.

### Moderators and mediators of treatment response

Better understanding of moderators of treatment effect defines *in whom* a treatment best works. Such information is important for “personalized treatment” of patients and for the proper allocation of resources. Better understanding of the mediators help explain *how* a treatment works. Understanding the mechanisms of action may be important for enhancing our ability to interpret clinical findings. If for example, we find little clinical impact of an “active” treatment and this were our only analysis, we would not be able to determine if the outcome were the result of “insensitive” outcome measures, a “weak” treatment, or a faulty design. However, if the treatment does in fact have significant effects on measures relevant to the proposed mechanism of action (e.g., changes in a relevant biological marker or psychological test), but is not accompanied by a change in clinical outcome, a strong argument can be made that the treatment “worked” on the proposed mechanism, but that it was not the appropriate mechanism to target.

While there is growing support for the use of rTMS in the treatment of TRMD, there are patient characteristics (e.g., patient age, gender, type of depression, and severity of psychiatric symptoms) that potentially moderate the treatment response. Several studies have shown that older patients have poorer outcomes after rTMS treatment compared to younger patients and younger patients also required fewer rTMS treatments to achieve remission [34]. It has also been suggested that gender may impact treatment response, as some studies have shown the antidepressant effect of rTMS to be greater in female than male subjects [35]. To date, most studies of rTMS have been conducted in those with unipolar depression, though there is some suggestion that participants with more complex presentations or severe psychiatric symptoms may evidence a different treatment response. Specifically, patients with psychotic depression and those with more severe symptoms of depression may experience significantly less improvement with rTMS treatment [36, 37]. These are important points for the current study as the typical Veteran with TRMD is a man in his mid-50s with a complex presentation of depression.

### Sample size and analysis plan

Based on the literature review, the study planning committee felt that a 10% difference between treatments would be of clinical relevance given the severity of the illness. With a sample size of 180 per group, the proposed study will have a power of 81% to detect an absolute difference between groups of 10% in the percentage of those participants who remit (6% sham and 16% rTMS). Thus, a total of 360 patients will be required from nine potential VA sites. Each site will be expected to randomize approximately 40 eligible patients over the recruitment period of the study.

All statistical tests will be two-sided, and tested at a 5% level of significance. All the statistical analyses will be conducted with SAS statistical analysis software, version 9.4 or later version (SAS Institute Inc., Cary, NC, USA). The primary hypothesis of the study is that there is a difference in the remission rate between the two interventions at the end of the acute treatment phase in VA patients with TRMD. The primary outcome measure in this study is success or failure to achieve remission from depression as defined by a score on the HRSD24 of 10 or less. The primary analysis will be done as an “intent-to-treat” analysis, i.e., patients will be analyzed in the groups to which they were randomized and dropouts will be considered treatment failures. The primary hypothesis will be addressed using a logistic regression model with PTSD diagnosis, history of substance use, and site as covariates. Site effects will be tested using logistic regression analysis examining the effect of treatment in a model that includes site and the interaction of site and treatment. Baseline comparability among the treatment groups will be evaluated with respect to such variables as demographics (e.g., age, gender, and race), baseline values of the outcome measures (e.g., the HRSD, quality of life (QOL) measure(s), suicidality, etc.), and the antidepressant currently being used, etc. The chi-square test and analysis of variance techniques will be used as appropriate to determine any differences in distribution of the variables across the treatment groups. Any variable that appears to be different between the groups (*p* < 0.10) will be evaluated to determine whether such imbalances had any effect on the conclusions.

In addition to the main analysis, using the entire randomized or intent-to-treat cohort, logistic regression models will be used for “completers” and also for “fully compliant” subjects to provide further information about treatment effects. For example, it would be expected that if rTMS had a significant clinical effect, its effect would appear greater in completer and totally compliant cohorts, than in the entire randomized cohort. Other analyses will be performed on secondary measures to further provide useful clinical information. Some secondary outcome measures, such as sustained response rate (“recovery”) and response on secondary outcome measures, can also be analyzed using logistic regression models. Other potential secondary analyses include change in suicidality, change in cognitive function, and change in QOL. The effect of rTMS on such measures will be determined using random regression and similar techniques that maximize the use of available data in repeated measures designs. At the end of the study, cost-effectiveness analysis will be performed.

The primary endpoint analysis will be based on the primary outcome measurement at the end of the treatment. Secondary outcome analyses are focused on the three time points, i.e., baseline, end of the treatment, and end of the follow up. However, the measurements from all time points will be used to illustrate the trend of outcome measurement changes across time.

Subjects who drop out during the treatment phase will be considered as treatment failures for the purpose of the primary analysis and the missing values will not be imputed. Multiple imputations will be used to handle missing data using Rubin’s method for secondary analyses.

## Publication of research results

The policy of the Cooperative Studies Program is that outcome data will not be revealed to the participating investigators until the data collection phase of the study is completed. This policy safeguards against possible biases affecting the data collection.

All presentations and publications from this study will follow CSP policy as stated in the CSP Guidelines. The presentation or publication of any or all data collected by participating investigators on patients entered into the Department of Veterans Affairs Cooperative Study is under the direct control of the study’s Executive Committee. This is true whether the publication or presentation is concerned with the results of the principal undertaking or is associated with the study in some other way. No individual participating investigator has any inherent right to perform analyses or interpretations or to make public presentations or seek publication of any or all of the data other than under the approval of the Executive Committee. The CSP also requires that every manuscript be reviewed and approved by the CSPCC Director prior to submission as a final quality control step.

Following completion of the study, a manuscript will be prepared for the primary outcome. This manuscript will describe the effect of rTMS on various measures of depressive symptoms. Additional manuscripts may be prepared to report on secondary outcome findings, including effects of rTMS on suicidality, cognitive function, and quality of life.

## Discussion

There is increasing literature demonstrating that rTMS may be a safe and effective treatment for TRMD, yet it is unclear if these benefits extend to the more complex Veteran population. Typically, patients with TRMD at the VA present with more psychiatric comorbidities such as PTSD, history of substance use, and suicidality. Traditionally, these complex cases have been excluded from both large industry and NIH studies; thus, the relevance of these prior findings to the VA patient with TRMD is limited. As the typical Veteran patient with TRMD is not likely to be seen in the ongoing rTMS trials, it is not known how typical VA patients will fare with rTMS. The proposed trial will be able to answer this question as it will include patients with some suicidal ideation; it will include typical VA patients with dual-diagnosis TRMD who may also present with PTSD and/or a history of substance use disorder, and will address issues of accessibility of care that often prevent effective treatment of TRMD and suicidal ideation in VA patients.

In addition to meeting a great need within the VA healthcare system, this trial also has numerous innovative techniques that are valuable for inclusion into other clinical trials. This is the first multisite study of rTMS to treat depression using the 6 cm rule for targeting, but the technique has been used in at least one other study of pain rTMS. The sham and active coils are integrated as one and deployed with a switch guided by the treatment number. Rather than exclude patients based on comorbid conditions, this study includes a vast amount of demographic data, which can serve to guide future studies in diverse patient groups. The goal will ultimately be to promote personalized treatment through the tailoring of brain stimulation based on the needs of the patient.

### Future work on mechanisms of action, genetics, and rTMS in Veterans

While the current study is addressing several important potential moderators of treatment response and proposed mechanisms of action, we acknowledge there are additional potential mechanisms of action. Response to treatment may depend on several genetic and biochemical moderators, including brain-derived neurotrophic factors (BDNF) [38], Apolipoprotein E (APOE) [39], serotonin transporter genes [40], and polymorphisms in Catechol-O-methyl transferase (COMT) [40]. While these targets are beyond the scope of the current trial, they are worthy of future investigation as an understanding of these moderators could help determine both the best course of treatment for an individual and the mechanisms of rTMS in cell signaling.

We also acknowledge that there will be groups of Veterans who do not qualify for this trial, but could potentially obtain great benefit from rTMS. These groups include those Veterans with more mild levels of depression and those Veterans with significant cognitive impairment due to various forms of dementing illnesses and the residual effects of traumatic brain injury (TBI). Thus, it will be important for future trials to include these groups in their design.

## Trial status

This trial has not completed patient recruitment at the time of submission.

### Additional files

Additional file 1: SPIRIT 2013 checklist: recommended items to address in a clinical trial protocol and related documents. (DOCX 49 kb)

Additional file 2: VA Research Consent Form. (PDF 326 kb)

Additional file 3: Data Management Security Plan. (DOCX 18 kb)

## Appendix A

**Table 4:** World Health Organization trial registration dataset

Data category	Information
Primary registry and trial identifying number	ClinicalTrials.gov NCT01191333
Date of registration in primary registry	26 August 2010
Secondary identifying numbers	None
Source(s) of monetary or material support	Cooperative Studies Program, Department of Veterans Affairs
Primary sponsor	Cooperative Studies Program, Department of Veterans Affairs
Secondary sponsor(s)	None
Contact for public queries	*Gerald Georgette, RN* 650-849-1942 Gerald.Georgette@va.gov
Contact for scientific queries	*Jerome Yesavage, MD* Phone: 650-852-3287 E-mail: yesavage@stanford.edu Department of Psychiatry Stanford University School of Medicine 3801 Miranda Avenue Palo Alto, CA 94304, USA
Public title	The Effectiveness of rTMS in Depressed VA Patients
Scientific title	The Effectiveness of rTMS in Depressed VA Patients
Countries of recruitment	USA
Health condition(s) or problem(s) studied	Repetitive Transcranial Magnetic Stimulation (rTMS) Treatment Treatment-Resistant Major Depression (TRMD)
Intervention(s)	Active comparator: *rTMS* Placebo comparator *Sham rTMS*
Key inclusion and exclusion criteria	*Inclusion Criteria* Between 18 and 80 years of age Using the Structured Clinical Interview for DSM Disorders (SCID) for DSM-IV-TR patients will be diagnosed MDD Have a HRSD24 ≥ 20 no more than 7 days prior to randomization Exhibit moderate level of resistance to antidepressant treatment defined, using the ATHF, as failure of at least two adequate medication trials Duration of current episode of MDD ≤ 10 years Ability to obtain a Motor Threshold (MT) (should be determined at the end of the screening process) Currently under the care of a VA psychiatrist If on a psychotropic medication regimen, that regimen will be stable for at least 4 weeks prior to randomization to the study and patient will be willing to remain on a stable regimen during the acute treatment phase Has an adequately stable condition and environment to enable attendance at scheduled clinic visits For female participants, agrees to use one of the following acceptable methods of birth control Able to read, verbalize understanding and voluntarily sign the Informed Consent Form prior to performance of any study-specific procedures or assessments *Exclusion Criteria* Pregnant or lactating female (this is an FDA-required exclusion; in the future, if rTMS becomes a proven treatment for major depression, its safety in the context of pregnancy should be studied separately) Unable to be safely withdrawn, at least two-weeks prior to treatment commencement, from medications that substantially increase the risk of having seizures Have a cardiac pacemaker Have an implanted device or metal in the brain Have a cochlear implant Have a mass lesion, cerebral infarct, increased intracranial pressure, or other active central nervous system (CNS) disease, including a seizure disorder Known current psychosis as determined by DSM-IV or SCID (axis I, psychotic disorder, schizophrenia) or a history of a non-mood psychotic disorder Known current bipolar I disorder as determined by SCID or a history of bipolar I disorder Current amnestic disorders, dementia, Blessed Orientation-Memory-Concentration > 10, delirium, or other cognitive disorders Current substance abuse (not including caffeine or nicotine) as determined by positive toxicology screen, or by history via SCID, within 3 months prior to screening Patients with an elevated risk of seizure due to TBI Participation in another concurrent clinical trial Patients with prior exposure to rTMS Active current suicidal intent or plan as evidenced by a score of 4 or 5 on the suicidal ideation portion of the Columbia Suicide Severity Rating Scale (CSSRS) or the endorsement of an actual attempt, interrupted attempt, or an aborted attempt in the past 6 months Unstable cardiac disease or recent (<3 months previous) myocardial infarction Patient refuses to sign consent for participation in the study
Study type	Interventional Allocation: randomized Intervention model: parallel assignment Masking: double blind Primary purpose: treatment Phase III
Date of first enrollment	June 2013
Target sample size	360
Recruitment status	Stopped
Primary outcome(s)	Proportion of subjects in each treatment group who achieve “remission” from depression at the end of the acute treatment phase
Key secondary outcomes	The key secondary outcomes include: Montgomery-Asberg Depression Rating Scales (MADRS), Beck Depression Inventory (BDI), Suicide Ideation measured by Beck Scale for Suicide Ideation (BSS), VR-36, and a neuropsychological battery

## Abbreviations

ADNI: Alzheimer’s Disease Neuroimaging Initiative
APOE: Apolipoprotein E
ATHF: Antidepressant treatment history form
BDI: Beck Depression Inventory
BDNF: Brain-derived neurotrophic factors
BHS: Beck Hopelessness Scale
BOMC: Six-Item Blessed Orientation-Memory-Concentration
BSS: Beck Scale for Suicide Ideation
CAPS: Clinician Administered PTSD Scale
CNS: Central nervous system
COMT: Catechol-O-methyl transferase
COWAT: Controlled Oral Word Association Test
CSP: Cooperative Studies Program
CSSRS: Columbia Suicide Severity Rating Scale
DAST: Drug Abuse Screening Test
DMC: Data Monitoring Committee
DSM-IV: *Diagnostic and statistical manual of mental disorders, 4* ^*th*^ *edition*
ECT: Electroconvulsive therapy
FDA: Food and Drug Administration
HRSD24: Hamilton Rating Scale for Depression
IRB: Institutional Review Board
JLO: Judgement of Line Orientation
LSC-R: Life Stressor Checklist-Revised
MADRS: Montgomery-Asberg Depression Rating Scale
MAOIs: Monoamine oxidase inhibitors
MAST: Michigan Alcohol Screening Test
MDD: Major depressive disorder
MT: Motor threshold
NAART: North American National Adult Reading Test
PCL: Posttraumatic Stress Disorder Checklist
PTSD: Posttraumatic stress disorder
QIDS-C16: Quick Inventory of Depressive Symptomatology
RAVLT: Rey Auditory-Verbal Learning Test
rTMS: Repetitive transcranial magnetic stimulation
SC: Study Coordinator
SCID: Structured Clinical Interview for DSM Disorders
SDMT: Symbol Digit Modalities Test
SF-36V: Short Form 36-Item Health Survey for Veterans
SI: Site Investigator
SMART: Site Monitoring, Auditing and Resource Team
STAXI-2: State Trait Anger Expression Inventory-2
TBI: Traumatic brain injury
THQ: Trauma History Questionnaire
TLFB: Alcohol/drug timeline follow-back
TMT: Trail Making Test
TRMD: Treatment-resistant major depression
UADE: Unanticipated Adverse Device Effects
VA: Veterans Affairs
VR-36: Veterans RAND 36-Item Health Survey
