# Supplementary material for: Repetitive transcranial magnetic stimulation (rTMS) for treatment-resistant major depression (TRMD) Veteran patients: study protocol for a randomized controlled trial
Source: Trials. 2017 Sep 2;18:409. doi: 10.1186/s13063-017-2125-y (PMC5581925; doi:10.1186/s13063-017-2125-y)
Supplement: Supplementary file 2 — VA Research Consent Form. (PDF 326 kb) [file 13063_2017_2125_MOESM2_ESM.pdf]

Participant Name: ____ Date:__________

Title of Study: _CSP # 556, "The Effectiveness of rTMS in Depressed VA Patients"_________

Principal Investigator: Jerome Yesavage, M.D.__________Facility: _Palo Alto VAMC______

MODEL TEMPLATE

INTRODUCTION

You are being invited to take part in a research study that is being funded by the Department of Veterans Affairs. Before you decide to take part, it is important for you to know four things. Why the research is being done. What it will involve. What the potential risks are. What the potential benefits are.

Read the information below closely. Discuss it with family and friends if you wish. Ask study staff about anything that is not clear or if you would like more details. Take your time to decide. If you do decide to take part, your signature on this consent form will show that you received all of the information below. It will also show that you were able to discuss any questions and concerns you had with a member of the study team.

You will be asked to answer questions (Attachment #1) about the information in this consent form to show that you understand it.

You will remain under the care of your primary VA psychiatrist before, during and after participation in this study.

TERMS

There are some terms you may need to know while reading this consent form:

rTMS (repetitive Transcranial Magnetic Stimulation): rTMS uses brief pulses of magnetic energy to stimulate nerve cells in the brain.

TRMD (Treatment Resistant Major Depression): Major Depression is a serious psychiatric illness. Some of the symptoms are feeling sad or blue, hopeless, helpless, and worthless. Other symptoms are problems sleeping, changes in appetite, guilt, and thoughts of death. TRMD is a type of depression where drugs have not worked very well.

Sham Treatment: In sham treatments, the doctor or nurse goes through the motions without actually treating. This will look, feel and sound like the real treatment but will not stimulate the brain. This is like using a placebo. A placebo is a pill that looks like a real pill but does not contain the real drug. The sham treatment will be used by about half the participants.

rTMS machine: An rTMS machine is a device that can deliver a high number of magnetic pulses per second. The magnetic pulses are delivered through coils that are encased in plastic. The machine consists of a computer console, much like a desktop computer, connected to a 'wand'. The wand is collection of wires wrapped in plastic. This wand is not magnetic when

there is no electricity going through it. When the machine sends electricity through the wand, this creates a powerful but temporary magnetic field that travels through skin and bones. During rTMS sessions, you sit in a comfortable chair next to the console, and the wand rests on your head. The wand is used to focus the magnetic pulses on certain parts of the brain.

BACKGROUND AND PURPOSE

- The purpose of this study is to find out if rTMS helps people with depression who have not been helped by medication or who have not been helped enough by medication. A magnetic coil will be placed on your head. This coil uses short pulses of magnetic energy to stimulate the part of your brain below the coil.
- There have been more than 70 research studies using rTMS, and some devices, including one similar to what is used in this study, are approved by the FDA for the treatment of depression. However, the device and treatment protocol as used in this study is still considered experimental. We hope to learn whether or not rTMS helps people who have major depression that has not been helped by drugs. You have been selected as a possible participant because you have depression that does not appear to have been helped by drugs.
- Three hundred and sixty veterans at around 9 VA Medical Centers across the United States will be in this study. About 40 will come from each medical center.
- This study will be conducted and sponsored by the Department of Veterans Affairs.

DURATION OF THE RESEARCH

The entire study will last about 3.5 years. You will be in the study about 39 weeks.

STUDY PROCEDURES

If you decide to take part in this study, this is what will happen. This study has 3 phases: screening (2-4 weeks), intervention (4-11 weeks), and follow-up (24 weeks).

1. SCREENING PHASE

If you agree to be in this study, you will complete a number of tests to make sure that you are healthy enough. You will read and sign this informed consent form before you begin the screening phase. The screening phase will take 7 to 8 hours to complete. It may be done in

MODEL TEMPLATE

one day or over several days. The screening phase will last between 2 and 4 weeks after signing the informed consent form.

During the screening phase and before you are given any rTMS treatments, the following will happen:

- You will be given a physical examination. A clinician will assess your medical history, and will ask questions about your mental health, your income and living situation, your mood, your current depressive symptoms and any feelings or thoughts of suicide.
- Study staff will review with you any drugs (prescriptions, "natural food products" and "over the counter") that you are taking or have taken in the past. During the study, you will not be able to take any drugs known to greatly increase the risk of seizures. Your primary VA psychiatrist will adjust your drugs as needed.
- You will complete several self-assessments about your mood (including thoughts of suicide), your health, your use of alcohol and other substances, and any possible traumatic experiences you may have had.
- You will work with study staff and your treatment team to complete a suicide safety plan prior to enrolling in the study. This is required of all participants.
- A blood sample will be taken to check how various systems in your body, like your liver and kidneys, are working. The total amount of blood in the sample will equal about 4 tablespoons.
- If you have a liver function test that is abnormal, you may need to return for additional tests.
- You will be asked to provide a urine sample. This sample will be screened for the use of drugs. Your urine screen results will not be disclosed to anyone outside this study but positive results may require that you be excluded from this study. If you are able to stop using these drugs, you may be re-screened later.
- You will have an alcohol test to measure your blood alcohol level. This will be for the screening of alcohol use. Your results will not be disclosed to anyone outside this study but positive results may require that you be excluded from this study. If you are able to limit your alcohol consumption, you may be re-screened later.
- You will be provided with the results of these blood, urine, and alcohol tests, if you request them.
- You will be tested with an rTMS coil in order to find the settings that will be used for your treatments. This is called a "motor threshold" and is the amount of magnetic power required to make your right thumb move by stimulating your brain. We will attach pads to your right thumb and hand with tape and non-permanent sticky glue. The pads will be

MODEL TEMPLATE

connected to a machine which measures the movement in your hand. We will use this machine, called an electromyograph or EMG, to find your motor threshold.

2. INTERVENTION PHASE

If you agree and are eligible to participate in this research study, you will be enrolled in the intervention phase of the study. This phase will last up to 11 weeks. You will come to the clinic for 20 to 30 sessions to receive your rTMS treatments. Each session will last around one hour. 25 minutes will consist of the actual rTMS treatment. There will normally be five daily sessions per week, Monday through Friday. After every fifth treatment, you will meet with study staff to complete study assessments that will last up to an additional hour. After the 20th session, you will be evaluated to determine if there has been any improvement in your depression. This will determine if any future sessions are needed. If you need additional sessions, you will receive either five or ten additional sessions. Your final session will require around 4 hours.

During the intervention phase, the following will happen:

- You will be randomized to either active "real rTMS" treatment or to sham treatment. In active treatment "real rTMS", brief pulses of magnetic energy are used to stimulate nerve cells in your brain. In sham treatment, the same machine is used but the nerve cells are not stimulated. Randomization is a process that is similar to flipping a coin where one side of the coin is active and the other side is sham. It is also similar to drawing a piece of paper out of a hat where some pieces say active and others say sham. There is a 50:50 chance of being randomized to either treatment group.

- All patients, regardless of whether they are getting active or sham TMS, will have mild electrical pads placed on the skin just underneath the TMS coil. During the TMS, there will be a slight electrical current passing through these pads, which will produce a mild tingling sensation. The purpose of this tingling is to make it hard to tell whether you are getting the active or sham TMS.

- Neither you nor your study doctor will know which treatment you are getting until the study is over. This type of study is called a double blind trial and this study type is being used so that your treatment and evaluation won't be affected by someone knowing whether or not you are getting active "real rTMS" or sham treatment. The study machine will know which treatment you are getting so that you will receive the same treatment at each visit. If your study doctor needs to know which treatment you are getting, he or she will be able to get that information.

MODEL TEMPLATE

- Before the first treatment, we will ask you whether you believe you will receive the active "real rTMS" or the sham (inactive) treatment. After the first treatment, we will ask you whether you believe you received the active "real rTMS" or the sham treatment.

- You will be retested to find your motor threshold on the first day of each of the 5-session blocks. You will be tested with an active coil to find the settings that will be used for you.

- You will be asked about any other drugs that you are taking and about side effects that may have occurred since your last visit. These may or may not be related to the study treatment. You will also be asked about the amount of alcohol or other substances you have consumed since your last visit. These questions will be asked at every session.

- You will be asked to provide a urine sample several times randomly during this phase. This sample will be screened for the use of drugs. Your urine screen results may be disclosed to your primary mental health provider if we think that you are using in a risky manner. You may also not be allowed to receive your rTMS treatment.

- You will have an alcohol test to determine your blood alcohol level several times randomly during this phase. This will be for the screening of alcohol use. Your results may be disclosed to your primary mental health provider if we think that you are using alcohol in a risky manner. You may also not be allowed to receive your rTMS treatment.

- You will be asked about your physical and mental health, your use of alcohol and other drugs, your mood, your current depressive symptoms and any thoughts or feelings of suicide.

- You will complete several self-assessments about how you are feeling after every 5th session.

The following is a description of the study procedure:

- You will be awake and alert throughout the treatment session.

- You will be seated in a chair. You will be provided with ear protection. Your head will be placed in a holder so that it is correctly positioned. You may close your eyes during treatment but not fall asleep.

MODEL TEMPLATE

- A metal coil in a plastic case will be held against the scalp on the left side of your head. You will hear a clicking noise as a few magnetic pulses are produced. The administrator will use the coil to find the area in your brain that causes your right thumb to move. This is called the Motor Threshold (MT).

- Participants normally notice only a loud clicking noise, and tingling sensation on the scalp. The coil may feel warm or hot against your head.

- Depending on the treatment group that you have been assigned to, you will receive either active "real rTMS" or sham (inactive) treatments.

- You may drive yourself to and from treatment sessions and attend to your normal daily tasks.

3. FOLLOW-UP PHASE

After the intervention phase of the study, you will enter a 24-week follow-up phase. If your depression has significantly improved during the intervention phase, you will receive 6 additional treatment sessions during the first three weeks (3 during the first week, 2 during the second, and 1 during the third) of the follow-up phase. During the follow-up phase, you will meet with study staff to complete study assessments. The amount of time required to complete each monthly visit (testing and evaluation) should be around 1 hour. The final follow-up visit will take about 4 to 5 hours. If you are unable to come in for a face to face follow-up visit, telephone visits may be arranged.

During the 24- week follow-up phase, the following will happen:

- Study staff will ask you about the following:
    - Any drugs that you are taking and side effects that may have occurred since your last visit.
    - Your physical and mental health, your mood and your current depressive symptoms.
    - Any thoughts or feelings of suicide.
- You will complete several self-assessments about your mood, your health, and any possible traumatic experiences you may have had.

MODEL TEMPLATE

- You will be asked to provide a urine sample several times during this phase. This sample will be screened for the use of drugs. Your urine screen results may be disclosed to your primary mental health provider if we think that you are using drugs in a risky manner.

- You will have an alcohol test to determine your blood alcohol level several times during this phase. This will be for the screening of alcohol use. Your results may be disclosed to your primary mental health provider if we think that you are using alcohol in a risky manner.

- At your final follow-up visit, we will ask you whether you believe you received the active "real rTMS" or the sham treatment.

4. FOR ALL STUDY PHASES

- Sleep is frequently disrupted when people are depressed. We recognize that you may have trouble sleeping. It is important for the treatment team to monitor the amount of sleep you get prior to each treatment session. If study staff believes that you have not gotten adequate sleep, they may cancel or reschedule that session.

- It is important for study staff to be aware of any changes in your medications during your participation in the study. If there are changes to your medications or you take them not as prescribed prior to a treatment session, study staff may choose to cancel or reschedule that session.

- You will interact with members of the entire study team. This includes a psychiatrist or neurologist, a nurse or physician assistant rTMS Operator, and a Study Coordinator. The study takes place at the (insert site name) during normal business hours, Monday through Friday, 8am to 4:30pm. If asked, we will provide a note for your employer that you were receiving medical treatment. We will not compensate for missed work time.

- You will be asked about adverse events whenever you are seen by study staff for treatment, evaluation, and follow-up visits. An adverse event is anything bad that happens with you and may or may not be related to your participation in this study. An independent committee will be told about all adverse events at least once every six months. If they believe that any aspect of this study is unsafe, they will recommend that changes be made to eliminate the safety problem.

MODEL TEMPLATE

FOR VA CENTRAL IRB USE ONLY

PI/SC Approval Date: 02/08/2016

LSI Approval Date: n/a

LSI Verification Date: n/a

RESPONSIBILITIES AND EXPECTATIONS OF STUDY PARTICIPANTS

In order to maximize the possible benefits of the rTMS treatment and to best ensure the safety of study participants, we will now go over the responsibilities and expectations of participation.

- Complete your questionnaires as instructed. You are free to skip any questions that you prefer not to answer.
- Ask questions as you think of them.
- Tell the investigator or research study staff if you think you might be pregnant.
- Tell the investigator or research staff if you change your mind about staying in the study.
- While participating in this research study, do not take part in any other research study without approval from the investigators. This is to protect you from possible injury from things such as extra blood drawing or potential drug interactions. Taking part in other research studies without approval from the investigators may invalidate the results of this research, as well as that of the other studies.
- Keep your study appointments. If it is necessary to miss an appointment, please contact the investigator or study staff to reschedule as soon as you know you will miss the appointment.
- It is important that you not give false, incomplete, or misleading information about your medical history, including past and present drug use, because this could have serious consequences for your well-being.
- The effects of alcohol and substance use while undergoing rTMS are not well known at this time. Alcohol use will be limited to 1 alcoholic beverage, defined as 12 oz. beer, 5 oz. wine, or 1.5 oz. hard liquor, a day. You cannot use illegal substances, such as marijuana, cocaine, and amphetamines, during your participation in the study. If you begin to use substances in a risky manner during your participation in this trial, study staff will notify your primary VA psychiatrist and you may be removed from the study. If you report consuming more than one alcoholic beverage or using substances prior to your treatment session, study staff may choose to cancel or reschedule that session.

MODEL TEMPLATE

Participant Name: ____ Date:__________

Title of Study: _CSP # 556, "The Effectiveness of rTMS in Depressed VA Patients"_________

Principal Investigator: Jerome Yesavage, M.D.__________Facility: _Palo Alto VAMC______

POSSIBLE RISKS OR DISCOMFORTS

Any procedure has possible risks and discomforts. The procedures in this study may cause all, some or none of the risks or side effects listed. Rare, unknown, or unforeseeable (unanticipated) risks also may occur. You need to carefully consider the following:

The drawing of blood may cause pain, bleeding, bruising, feeling faint and, on rare occasions, infection at the site of the needle insertion. Precautions will be taken to minimize these risks. The total amount of blood that you will be asked to give during the study is about 4 tablespoons.

If you are taking any drugs that may increase the risk of having a seizure, you will need to be taken off those drugs before you can participate. You and your physician will need to discuss the feasibility of your discontinuing any such medication. Withdrawal from such drugs may cause discomfort or illness.

A few patients receiving rTMS have had seizures. All of the reported seizures resolved promptly on their own and none had any lasting effects or adverse impact on the patients. There is little evidence of risk of seizures using rTMS the way it will be used in this study.

There may be an increased risk of seizures from combining the use of bupropion and rTMS.

In the unlikely event that a seizure does occur, you will be closely monitored and treated for any medical or psychological consequences. Lab tests will be drawn and you will be seen by a neurologist as soon as possible. The rooms where the rTMS studies are performed are fully equipped to safely handle a seizure. After the neurologist has seen you and determined what caused your seizure, you will be given a letter regarding the seizure to share with your primary health care provider. If you have no other medical or neurological problem that caused the seizure, the letter will indicate that the seizure during rTMS does not increase your risk for future seizures.

rTMS treatment can result in mild to moderate headaches in as many as 30 out of 100 of patients. Some people also report discomfort at the site of rTMS stimulation. This occurs in around 15 out of 100 of patients. Headaches and site discomfort usually readily respond to acetaminophen or ibuprofen. Painfulness improves over time or goes away. Often patients fall asleep in the second week while receiving the same treatment that on the first day was reported as very painful.

MODEL TEMPLATE

FOR VA CENTRAL IRB USE ONLY

PI/SC Approval Date: 02/08/2016

LSI Approval Date: n/a

LSI Verification Date: n/a

There is a small risk of dental pain with rTMS, during or immediately after the treatment. If this occurs, let your study doctors and nurses know and they may be able to move the rTMS coil position or provide you with a bite block to reduce this pain or make it not happen.

rTMS treatment may produce movement or tingling of the arm, leg, face or scalp. You may also experience a temporary feeling of numbness in the face.

During treatment the coil may get warm. It may feel about the same as a heating pad on low or medium setting. This may be uncomfortable but should not be painful.

There is a possible risk of hearing loss due to the sounds made by the device. You will wear earplugs and headphones during your rTMS sessions. This should greatly reduce the possibility of hearing loss. If you think your hearing is getting worse during the study, tell the study team right away. After your last study treatment, you may keep the headphones if you choose.

The rTMS operator will monitor you for ear protection, coil placement, and seizure activity during all sessions.

In some people, daily prefrontal rTMS can cause them to have increased energy, no need for sleep, and rapid racing thoughts. This is called mania. If you notice these changes let your primary mental health provider and study team know.

A major risk in treating seriously depressed patients is the risk of suicide. We will work with you and your primary mental health provider in the creation of a written safety plan prior to your participation in the study. One part of the safety plan may be the requirement that all firearms either be removed from your residence or be placed under lock and key, including trigger locks, with guns and ammunition locked separately and the keys given to another family member or friend.

You will frequently be asked about "suicidal thoughts" during the study. This is not because we think the treatment will make you suicidal, but rather because we know that you are depressed and many depressed people think of suicide. Please give honest and open answers to such questions and we will try to help you get over any such feelings. And because this is such an important issue, if you have any suicidal thoughts, it is vital that you seek appropriate care immediately. An actual suicide attempt will result in not being able to continue study treatments and you will immediately enter the 24 weeks (6 month) follow-up phase.

MODEL TEMPLATE

Your study investigator will be monitoring you during your participation to see if you are experiencing any side effects. It is important that you report promptly any side effect to study staff. If you feel, or your study investigator feels, that the side effects are not well tolerated, treatment may be stopped altogether and you may be withdrawn from the study.

The possibility of long-term risks is unknown. In previous studies, animal and human brains have shown no evidence of any kind of damage from rTMS. As with any experimental treatment, there may be unforeseen risks associated with this device. You will be informed of any new information that is developed during the study that might affect your willingness to continue your participation.

You will also be evaluated for current and previous medical and psychiatric diagnoses. You will be asked to report your use of alcohol and other substances (marijuana, cocaine, heroin, etc). You will also be asked to complete questionnaires that ask about your life satisfaction, quality of life, work, suicide ideation and other aspects of your life, as well as an interview about symptoms of depression. These questionnaires take around 5-30 minutes each to complete (total time, around 8 hours). The type, frequency, and intensity of your major depression symptoms will be evaluated during a 2 hour interview. The total time required for completing questionnaires, assessments, and interviews is around 5-10 hours and will be done over several visits. These questions may bring on uncomfortable thoughts, feelings, and lead to recalling troubling memories. In some cases the subject of questions and length may cause fatigue, discomfort, and/or boredom. It is important to remember that these questions are to be answered at your own pace. If you feel anything described above let the study coordinator know and he/she can continue the questions another day.

For Women of Child-bearing Potential
For safety reasons, pregnant women will not be allowed to participate in this study. This is because the effects of rTMS on an unborn child are not known. There may be unforeseeable (unanticipated) risks to the participant (or to the unborn child) if the participant is pregnant or becomes pregnant during the study.

You will have a urine pregnancy test within 7 days prior to your starting study treatment. Thereafter, you will have a urine pregnancy test every four weeks through the end of the study to be sure that you are not pregnant.

You must agree to use a medically acceptable form of birth control while participating in the study. Acceptable forms of birth control are:

- Complete abstinence (not having sexual intercourse with anyone)

MODEL TEMPLATE

- An oral contraceptive (birth control pills)
- Norplant
- Depo-Provera
- A condom with spermicide
- A cervical cap with spermicide
- A diaphragm with spermicide
- An intrauterine device
- Surgical sterilization (having your tubes tied)

If you become pregnant during the intervention phase of the study, you will not be able to continue the study treatments and you will immediately enter the 24 week (6 visits) follow-up phase. You will also be referred to a Women's Health Clinic. If you become pregnant during the follow-up phase of the study, you will continue to come in for all remaining follow-up phase visits and will complete all assessments as you normally would.

If you become pregnant at any time during the study, you will be asked to sign a release of information form for study staff to access medical records to obtain information regarding the outcome of your pregnancy. No pediatric records will be reviewed.

There is no likely effect on sperm count or the motility of sperm or other reproductive risks associated with fathering a child, although this has not been formally tested in humans. Likewise, there are no known risks on sperm and ova (eggs).

Risks of the usual care you receive are not risks of the research. They are not included in this consent form. You should talk with your health care providers about risks of usual care.

POTENTIAL BENEFITS

We can't promise that you will get any benefits from taking part in this research study. However, possible benefits may include relief from depression and improvement in quality of life. The information that is obtained during this study may be scientifically useful and may lead to greater knowledge about the treatment of depression.

MODEL TEMPLATE

Participant Name: ____ Date:__________

Title of Study: _CSP # 556, "The Effectiveness of rTMS in Depressed VA Patients"_________

Principal Investigator: Jerome Yesavage, M.D.___________Facility: _Palo Alto VAMC______

The medical testing done in this study could reveal a medical condition that you might not have previously been aware of and for which you may need treatment. Study staff will refer you for additional treatment if such problems are identified but the study will not pay for the treatment of any such identified problems.

ALTERNATIVE PROCEDURES

You may choose not to participate in this study. If this is your decision, there are other choices including the standard treatments provided by the local clinic. Your study investigator or a study clinician will discuss any alternatives with you before you agree to participate in this study. Alternative treatments include talk therapy, antidepressant drugs, rTMS treatment outside of the study, and electroconvulsive therapy (ECT). ECT is a medical treatment for severe mental illness in which a small, carefully controlled amount of electricity is introduced into the brain to cause a seizure. It is also known as "electrotherapy" or "shock therapy". You may also discuss these options with your doctor.

CONFIDENTIALITY

A description of this clinical trial will be available on http://www.ClinicalTrials.gov as required by U.S. Law. This Web site will not include information that can identify you. At most, the Web site will include a summary of the results. You can search this Web site at any time.

The information collected for this study will be kept confidential. We will include information about your study participation in your medical record. We will not share your study records or identify you except as described in this informed consent document. There are times when we might have to show your records to other people. For example, someone from the Office of Human Research Protections, the Government Accountability Office, the Office of the Inspector General, the VA Office of Research Oversight, the VA Central IRB, our local Research and Development Committee, and other study monitors may look at or copy portions of records that identify you.

We have obtained a Certificate of Confidentiality from the Federal Government. This helps protect your privacy by allowing us to refuse to release your name or other information outside of the research study, even by a court order. The Certificate of Confidentiality will not be used to prevent disclosures to local authorities of child or elder abuse and neglect, harm to self or others, or if we become aware that you have an infectious disease that State or Federal Law requires us to report. If we learn of such a situation, we are mandated to act appropriately, which may include revealing your identity as a research participant to authorities. The

MODEL TEMPLATE

FOR VA CENTRAL IRB USE ONLY

PI/SC Approval Date: 02/08/2016

LSI Approval Date: n/a

LSI Verification Date: n/a

Certificate does not prevent you or a member of your family from releasing data about yourself or your involvement in this study.

During this research study we will use personal and health information for the scientific goals of the study. The information collected for this study will be kept confidential except where disclosure is required by law. For example, if you appear to want to do harm to yourself (suicide) or to others, we will report this information to the appropriate authorities and assist you in obtaining care. We may also contact your primary mental health provider regarding clinically significant status changes. All local, state and federal regulations will be followed when releasing study data. Any reports or publications resulting from this study will not include any information that could identify you.

We will use your SSN to access VA databases to extract information about your use of VA health care services outside of the trial, including those provided by non-VA providers that the VA pays for, and the costs of these services. This includes records on all of the medicines that you receive from the VA. Your SSN will be matched to the scrambled SSN that the VA uses as a patient identifier in these datasets. Your actual SSN will only be used to obtain the scrambled SSN; the real and scrambled SSNs will never be in the same data file and the real SSN will be in an encrypted file except for when we use it to link to the scrambled SSN.

Your social security number and name will be kept separate from all of your study data. In signing this informed consent you authorize the use of your social security number and last name for administrative access to the databases described above. You may not participate in this study if you are not willing to give us your social security number.

Data collected during the study will be stored in a way that does not identify you by name. All data forms and reports will be coded. Research and clinical records will be stored in a locked cabinet. Only selected study researchers will have access to this information. They are bound by rules of confidentiality not to reveal identifying information to others. All data collected for this study will be sent electronically via a secure fax and/or online server to the VA Cooperative Studies Program Coordinating Center (CSPCC), Perry Point, Maryland and will be kept in a secure database. The CSPCC will be responsible for the processing and analyses of all research data. The Chairman’s Office (located at VAMC Palo Alto, CA), the Cooperative Studies Program Clinical Research Pharmacy Coordinating Center in Albuquerque, NM and members of the Executive Committee and the Data Monitoring Committee, as well as monitoring bodies associated with the study will review research data. Study records will be kept for the length of time required by law after the study is completed.

MODEL TEMPLATE

FOR VA CENTRAL IRB USE ONLY

PI/SC Approval Date: 02/08/2016

LSI Approval Date: n/a

LSI Verification Date: n/a

Participant Name: ____ Date:__________

Title of Study: _CSP # 556, "The Effectiveness of rTMS in Depressed VA Patients"_________

Principal Investigator: Jerome Yesavage, M.D.__________Facility: _Palo Alto VAMC______

Authorized personnel from the VA will see your medical records and the consent form that you signed. Other federal agencies such as the Food and Drug Administration (FDA) and other Federal agencies; e.g., the Office for Human Research Protection (OHRP) and the Government Accountability Office (GAO), the Office of the Inspector General, the VA Office of Research Oversight, the VA Central IRB, our local Research and Development Committee, and other study monitors may review your records to make sure that they meet federal, state or local regulations. Because of the need to allow access to your medical records by these agencies, absolute confidentiality cannot be guaranteed but every effort will be made to keep information about you both private and confidential.

You will not be able to have access to the research data that has been collected about you during the study. However, after the study is completed, which is after last participant has completed their follow-up, you will be notified which treatment you received during the study.

By signing this informed consent form, you are giving us permission to use the information collected about your health only until the end of the study. You have the right, at any time, to take back your permission to use your personal health information for research purposes. However, if your information has already been sent to the Perry Point Cooperative Studies Program Coordinating Center or has been combined with other participants' information (such as when numbers are averaged) it will continue to be used. No further information about you will be collected. When your information is combined with other participants' information in the study, your personal information cannot be identified.

If you have any questions about withdrawing your permission, you may contact [insert name] at [insert phone number]. To withdraw your permission for the use of your personal health information, you must contact Dr. [insert name of PI] in writing at [insert address]. If you withdraw permission or do not give your permission, you will still receive all the medical care and benefits for which you are otherwise eligible but you will be unable to continue in this research study.

COSTS TO PARTICIPANTS AND PAYMENT

Costs to Participants

You, your insurance company or any other third party payer will not be billed for any study-related treatments, blood or urine tests or other procedures that are part of this study and not part of your routine treatment. If you receive treatment that is part of your usual care, you may be billed as you usually are.

FOR VA CENTRAL IRB USE ONLY

PI/SC Approval Date: 02/08/2016

LSI Approval Date: n/a

LSI Verification Date: n/a

MODEL TEMPLATE

For veterans who are required to pay co-payments for medical care and services provided by VA, these co-payment requirements will continue to apply for medical care and services provided by VA that are not part of this study.

Payment Offered for Participation

You will be compensated for your time and inconvenience. You will be responsible for transportation to and from all treatment and follow-up sessions.

You will be paid for your time and inconvenience in each of the three study phases as follows:

- Screening Phase: $40
- Intervention Phase: $300
- Follow-up Phase: $60

If you withdraw or stop early in any of the three phases, you will be paid according to what phase you are in. For example, if you withdraw at any time during the Intervention Phase you would receive payment of $40 for the screening phase and $300 for the Intervention Phase, but not $60 for the follow-up phase. If you complete all three phases you would receive a total of $400.

MEDICAL TREATMENT AND COMPENSATION FOR INJURY

Every reasonable safety measure will be used to protect your well-being. If you are injured as a result of taking part in this study, the VA will provide necessary medical treatment at no cost to you. Financial compensation is not available for such things as lost wages, disability or discomfort due to an injury.

If you should have a medical concern or get hurt or sick as a result of taking part in this study, call:
DURING THE DAY:

Dr./Mr./Ms. ___________________________ at ________________________ and

AFTER HOURS:

Dr. /Mr. /Ms.___________________________ at ________________________.

Emergency and ongoing medical treatment will be provided as needed.

MODEL TEMPLATE

FOR VA CENTRAL IRB USE ONLY

PI/SC Approval Date: 02/08/2016

LSI Approval Date: n/a

LSI Verification Date: n/a

Participant Name: ___________________________________ Date:__________

Title of Study: _CSP # 556, "The Effectiveness of rTMS in Depressed VA Patients"_________

Principal Investigator: Jerome Yesavage, M.D.__________Facility: _Palo Alto VAMC______

You do not give up any of your legal rights and you do not release the VA from any liability by signing this form.

In case of an emergency in which you are unable to reach [insert name of PI at (add contact information)], please call 911 or go to the nearest emergency room.

No promises have been given to you since the results and the risks of a research study are not always known in advance. However, every reasonable safety measure will be taken to protect your well-being. You have not released this institution from liability for negligence.

VOLUNTARY PARTICIPATION

Your participation is voluntary. It is up to you to decide whether or not to take part in this study. If you do not wish to be in this study or leave the study early, you will not lose any benefits to which you are otherwise entitled and still receive all usual care that is available to you. Your decision not to take part will not affect the relationship you have with your doctor or other staff and it will not affect the usual care that you receive as a patient.

If you decide to take part you may still withdraw your consent at any time and stop participation without penalty or loss of benefits. You are not waiving any legal claims, rights or remedies because of your participation in this research study. If you leave the study early for any reason, it is important to come in for a final study visit to ensure appropriate follow-up care outside of this research study.

For data already collected prior to your withdrawal, the investigator may continue to review the data already collected for the study but cannot collect further information, except from public records, such as survival data. Specimens already used cannot be withdrawn.

RIGHT OF INVESTIGATOR TO TERMINATE PARTICIPATION

At the discretion of the study team you may be withdrawn from this study.

Possible reasons for withdrawing you from the study include:

- You fail to follow instructions.
- You drink more than one glass of alcohol a day, defined as 12 oz. beer, 5 oz. wine, or 1.5 oz. hard liquor
- You abuse illegal drugs.

MODEL TEMPLATE

FOR VA CENTRAL IRB USE ONLY

PI/SC Approval Date: 02/08/2016

LSI Approval Date: n/a

LSI Verification Date: n/a

Participant Name: ______________________________ Date:__________

Title of Study: _CSP # 556, "The Effectiveness of rTMS in Depressed VA Patients"_________

Principal Investigator: Jerome Yesavage, M.D.__________Facility: _Palo Alto VAMC______

- You abuse or misuse prescription drugs.
- You become pregnant.
- The investigator decides that continuation could be harmful to you.
- You need treatment not permitted for participation in the study.
- The study is cancelled.
- Other administrative reasons.
- Unanticipated circumstances.

If you leave the study early for any reason, it is important to come in for a final study visit to ensure appropriate follow-up care outside of this research study.

PERSONS TO CONTACT

If you have any questions, complaints, and concerns about the research or related matters, you may contact _____________, the participating investigator at ____________________, _____ __________________, the study coordinator at ____________________, or the Patient Advocate of the [insert Medical Center name here] at __________________________.

If you have questions about your rights as a study participant, or you want to make sure this is a valid VA study, you may contact the VA Central Institutional Review Board (IRB). This is the Board that is responsible for overseeing the safety of human participants in this study. You may call the VA Central IRB toll free at 1-877-254-3130 if you have questions, complaints or concerns about the study.

SIGNIFICANT NEW FINDINGS

Sometimes during the course of a research study, new information becomes available about the treatment that is being studied that could change your willingness to continue in the study. If this happens, your research doctor will tell you about it and discuss with you whether you want to continue in the study. If you decide to withdraw at that time, your research doctor will make arrangements for your medical care to continue. If you decide to continue in the study, you may be asked to sign an updated consent form. Your research doctor could also decide that it may be in your best interests to withdraw you from the study. He/she will explain the reasons and arrange for your medical care to continue.

MODEL TEMPLATE

FOR VA CENTRAL IRB USE ONLY

PI/SC Approval Date: 02/08/2016

LSI Approval Date: n/a

LSI Verification Date: n/a

Participant Name: ____ Date:__________

Title of Study: _CSP # 556, "The Effectiveness of rTMS in Depressed VA Patients"_________

Principal Investigator: Jerome Yesavage, M.D.___________Facility: _Palo Alto VAMC______

AGREEMENT TO PARTICIPATE IN THE RESEARCH STUDY

Dr./Mr./Ms. __________________________ has explained the research study to you. You have been told of the risks or discomforts and possible benefits of the study. You have been told of other choices of treatment available to you. You have been given the chance to ask questions and obtain answers.

You voluntarily consent to participate in this study. You also confirm that you have read this consent, or it has been read to you. You will receive a copy of this consent after you sign it. A copy of this signed consent will also be put in your medical record if applicable.

**I agree to participate in this research study as you have explained in this document.**		
___________________________<br>Participant's Name	___________________________<br>Participant's Signature	___________<br>Date
______________________________<br>Name of person obtaining authorization and consent	________________________________<br>Signature of person obtaining authorization and consent	_____________<br>Date

MODEL TEMPLATE

FOR VA CENTRAL IRB USE ONLY

PI/SC Approval Date: 02/08/2016

LSI Approval Date: n/a

LSI Verification Date: n/a

Participant Name: ______________________________ Date:__________

Title of Study: _CSP # 556, "The Effectiveness of rTMS in Depressed VA Patients"_________

Principal Investigator: Jerome Yesavage, M.D.__________Facility: _Palo Alto VAMC______

ATTACHMENT 1 - CONSENT FORM QUESTIONS
The Effectiveness of rTMS in Depressed VA Patients

		TRUE	FALSE
1)	Your participation in this research study is voluntary.	☐	☐
2)	There are no potential risks or side effects associated with the use of this experimental device in this research study.	☐	☐
3)	Your participation in the study may last up to 21 weeks.	☐	☐
4)	You will not have to give any blood or urine samples at any time during the course of the study.	☐	☐
5)	Your participation in the study will be kept confidential except as required by law.	☐	☐
6)	The study staff may end your participation in this study if they feel that to do so would be in your best interest.	☐	☐
7)	You will be compensated during this trial for completing all required tests and study assessments.	☐	☐
8)	A woman who becomes pregnant during the intervention phase of the study may continue to receive rTMS treatments and will not be terminated from the study.	☐	☐
9)	You do not have to inform the study staff of any new medicines that you take during the study.	☐	☐
10)	You will receive active "real rTMS" treatment.	☐	☐
11)	After your final follow-up visit, you will not receive further rTMS treatment as a part of the study.	☐	☐

The correct answers to the questions above have been discussed with me.

____

Participant's Signature Date

FOR VA CENTRAL IRB USE ONLY

PI/SC Approval Date: 02/08/2016

LSI Approval Date: n/a

LSI Verification Date: n/a

MODEL TEMPLATE
