# Supplementary material for: Repetitive transcranial magnetic stimulation (rTMS) for treatment-resistant major depression (TRMD) Veteran patients: study protocol for a randomized controlled trial
Source: Trials. 2017 Sep 2;18:409. doi: 10.1186/s13063-017-2125-y (PMC5581925; doi:10.1186/s13063-017-2125-y)
Supplement: Supplementary file 3 — Data Management Security Plan. (DOCX 18 kb) [file 13063_2017_2125_MOESM3_ESM.docx]

**Additional file 3. Data Management Security Plan**

Data Collection and Data Entry

Based on source documents collected at the study sites, data will be collected and then entered at the site using electronic data capture (EDC). The VA Cooperative Studies Program Coordinating Center (CSPCC) at Perry Point will develop the EDC templates. CSPCC will function as the centralized data management center for the study. The medical record, laboratory reports and all related documents will be the source of verification of data entered. Data should be entered on an ongoing and regular basis throughout the study and in accordance with the instructions in the study operations manual. The SI is responsible for maintaining accurate, complete and up-to-date records for each participant. The SI is also responsible for maintaining any source documentation related to the study, including any films, tracings, computer discs or tapes.

CSPCC will be responsible for the validation of the clinical database, ensuring data integrity, and for the training of all participating staff on applicable data management procedures. InfoPath will be utilized in this clinical trial. Any discrepancies (i.e., missing data, range validation, cross check) that are discovered during the verification process will be flagged with quality control notes and clinical sites will be required to either correct or confirm flagged entries. The CSPCC will send Quality Control Reports to the Chairman’s Office and to the participating sites on a monthly basis. These reports will summarize the quality and quantity of the data that each site has submitted.

When the study is completed and all data have been entered into the clinical database and the database has been checked for quality assurance and is locked, the CSPCC statisticians, in accordance with the Analytical Plan Section of this protocol, will perform statistical analysis of the data. Periodically, during the study, CSPCC will prepare various types of summary reports of the data so that progress of the study can be monitored. These reports will be prepared for the Data Monitoring Committee (DMC) and others, as appropriate.

Study Documentation and Records Retention

Study documentation includes all CRFs, quality control notes, workbooks, source documents, monitoring logs and appointment schedules, sponsor-investigator correspondence and regulatory documents (e.g., signed protocol and amendments, IRB correspondence and approved consent form and signed informed consent forms, Statement of Investigator form, and clinical supplies receipt and distribution records).

Source documents include all recordings of observations or notations of clinical activities and all reports and records necessary for the evaluation and reconstruction of the study. Thus, source documents include, but are not limited to laboratory reports, audiology reports, patient diaries and progress notes, hospital charts or pharmacy records and any other reports or records of any procedure performed in accordance with the protocol.

Whenever possible, the original recording of an observation should be retained as the source document; however, a photocopy is acceptable provided that it is a clear, legible, and exact duplication of the original document.

Research records for all study participants including medical history and physical findings, laboratory data, and results of consultations with the primary care VA psychiatrist are to be maintained by the investigator in a secure storage facility for 3 years after the end of the study or until notified by CSPCC. These records are to be maintained in compliance with IRB, State and Federal requirements, whichever is longest. Exceptions to the 3-year retention requirement can be found in 45 CFR 74.53 and 92.42 (e.g., if any litigation, claim, financial management review, or audit is started before the expiration of the 3-year period, the records must be retained until all litigation, claims, or audit findings involving the records have been resolved and final action taken). It is the investigator’s responsibility to retain copies of the completed CRFs until notified in writing by CSPCC that they can be destroyed. In all instances, the site must get permission from CSPCC prior to disposition of any study documentation and materials.

All records with identifiers will be stored indefinitely in accordance with the VA Records Control Schedule.

Data Security Plan

All data collected for this study will be handled and used in compliance with both the CSP and the Perry Point CSPCC data security plans. All patient level data will be treated as protected health information. Data will be transmitted from participating sites using secure servers. Study personnel at CSPCC, CRPCC, and at participating sites will be required to complete annual training courses. These courses will cover good clinical practices, human subjects’ protection, cyber security, and privacy policy. Any data security breaches will be immediately reported. Access to patient level data at CSPCC or CRPCC, will be obtained through user accounts which will be protected by strong passwords. File protections will be used to limit access to members of the study group. Patient level data will never be stored on portable storage devices unless it is encrypted, explicitly authorized, and use specific.
